# Primary vaginal Ewing sarcoma with uterine fibroid: A case report

**DOI:** 10.1097/MD.0000000000020859

**Published:** 2020-07-02

**Authors:** Maolin Xu, Yulin Liu, Shue Zeng, Hongbin Wang, Gaolong Weng, Fang Li

**Affiliations:** a Department of Radiology; b Department of Ultrasound; c Department of Gynecological Oncology, Hubei Cancer Hospital, Tongji Medical College, Huazhong University of Science and Technology, Wuhan, Hubei Province, China.

**Keywords:** Ewing sarcoma, uterine fibroid, vagina

## Abstract

**Rationale:**

Extra osseous Ewing sarcoma (ES), an uncommon malignant neoplasm, accounts for about 15% of Ewing sarcoma, which mainly affects paravertebral region, lower extremity, chest wall, retroperitoneum, pelvis, and hip. Here is a 54-year-old woman of primary vaginal Ewing sarcoma with uterine fibroid, which has been fewly known or reported.

**Patient concerns:**

The patient was admitted to our hospital because of vaginal pain. Her uterus showed as parallel position and enlarged as about 3 months of pregnancy size.

**Diagnosis:**

Magnetic resonance imaging (MRI) and ultrasonography (US) demonstrated 2 heterogeneous masses in the vagina and uterus, respectively. Ultrasound-guided puncture biopsy revealed a malignant tumor in the right lateral vaginal wall.

**Interventions:**

The patient was treated by hysterectomy, bilateral salpingo-oophorectomy, and tumors excision, with the subsequent treatment of chemotherapy.

**Outcomes:**

The patient recovered well without local recurrence for >1 year.

**Lessons:**

Primary vaginal Ewing sarcoma is extremely rare. The treatments of uterine fibroid include uterine artery embolization and surgical options, While wide local excision followed by adjuvant chemotherapy and/or radiotherapy should be recommended for the vaginal ES.

**Highlights**

- A 48-year literature review from 1970 to 2018 revealed that <30 cases of primary vaginal Ewing's sarcoma have been previously reported, from which few had discussed the imaging findings about primary vaginal Ewing sarcoma.
- Vaginal ES and uterine fibroid have certain manifestations and imaging features in MRI and US, but not specific.
- In addition, transvaginal ultrasound-guided puncture biopsy from the vaginal mass revealed the diagnosis of Ewing sarcoma.

## 1 Introduction

As a highly malignant neoplasm of bones, Ewing sarcoma (ES) usually occurs during childhood, and nearly 15% of ES are extraosseous.^[1]^ Extraosseous Ewing sarcoma (EES) grows rapidly, with round-cell malignancy of uncharacterized mesenchymal cell origin.^[2]^ Virtually, all Ewing sarcomas share a common chromosomal translocation of the long arm of chromosome 11 and 22. The translocation of the EWSR1 gene on to chromosome 22p12 next to the FLI1 gene may cause upregulation of insulin-like growth factor 1, playing a key role in cellular proliferation.^[3]^ The common translocation and a strong membranous expression of CD99 could unify the diagnosis of extraosseous Ewing sarcoma.^[4]^ A 48-year literature review from 1970 to 2018 revealed that <30 cases of primary vaginal Ewing's sarcoma had been previously reported,^[1,2,4]^ and few literatures had discussed the imaging findings of primary vaginal Ewing sarcoma. In addition, uterine fibroid is the most commonly benign tumor in female pelvis, and occurs in about 20% to 50% of women around the world, from which black women of reproductive age have the highest incidence.^[5–7]^ We present a rare case of a 54-year-old woman with primary vaginal Ewing sarcoma accompanying with uterine fibroid. Lesions were detected by ultrasonography (US) and magnetic resonance imaging (MRI) examination.

## 2 Case report

A 54-year-old woman was admitted to our hospital for the pain in the vagina. She had been with the symptom for about 4 days. Upon gynecological examination, the vaginal tumor had the following characteristics: painful mass, indurated and immobile, with the size of 6.5 × 6.0 × 4.0 cm. The mass lesion arised in the right lateral vaginal wall, extending to labia majora. Her uterus showed as parallel position and enlarged as about 3 months of pregnancy size. Cervix and all the vaginal fornices were free from mass.

**Figure 1:**
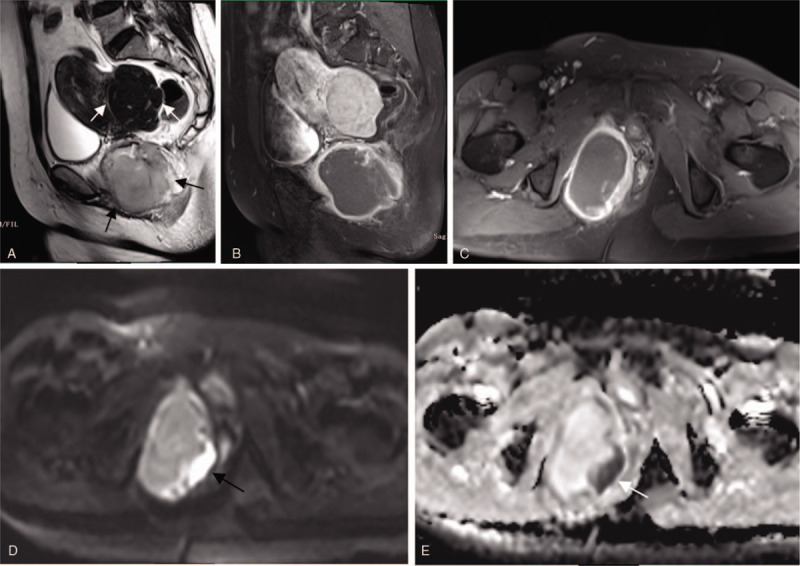
MR examination of the pelvis. (A) Sagittal T2-weighted image showed a 7.5 × 4.9 cm solid and cystic mass with obscure margin (black arrow) and a 6.7 × 4.8 cm smooth, well-defined mass (white arrow). (B) Sagittal T1-weighted-enhanced image showed that the uterine tumor appears as heterogeneous enhancement, in the posterior uterine wall. (C) Axial T1-weighted-enhanced image showed that the vaginal tumor's edge appears as marked enhancement, in the ischiorectal fossa adjacent to the right lateral vaginal wall. (D) Axial diffusion-weighted image and (E) apparent diffusion coefficient image showed that the vaginal tumor's edge appears as restricted diffusion (black and white arrow).

MRI images revealed a 7.5 × 4.9 cm solid and cystic mass, which was identified in the ischiorectal fossa adjacent to the right lateral vaginal wall. The mass had obscure margin, while lesion's edge showed both restricted diffusion and marked enhancement. However, the location of the mass was considered to be indeterminate on MRI. Additionally, the uterine mass, measured 6.7 × 4.8 cm, had smooth and well-defined margin, but with inhomogeneous enhancement (Fig. 1A–E).

**Figure 2:**
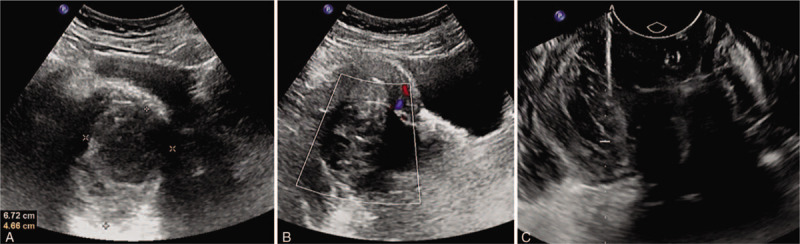
US examination of the pelvis. (A) Color Doppler showed a 6.72 × 4.66 cm hypoechoic or anechoic mass, with no obvious blood flow signal. (B) Color Doppler showed a 6.26 × 5.00 cm well-defined heterogeneous hypoechoic mass in the medial aspect of posterior uterine wall, with a little blood flow signal in peripheral of mass. (C) Transvaginal ultrasound-guided puncture biopsy from vaginal mass. US = ultrasonography.

**Figure 3:**
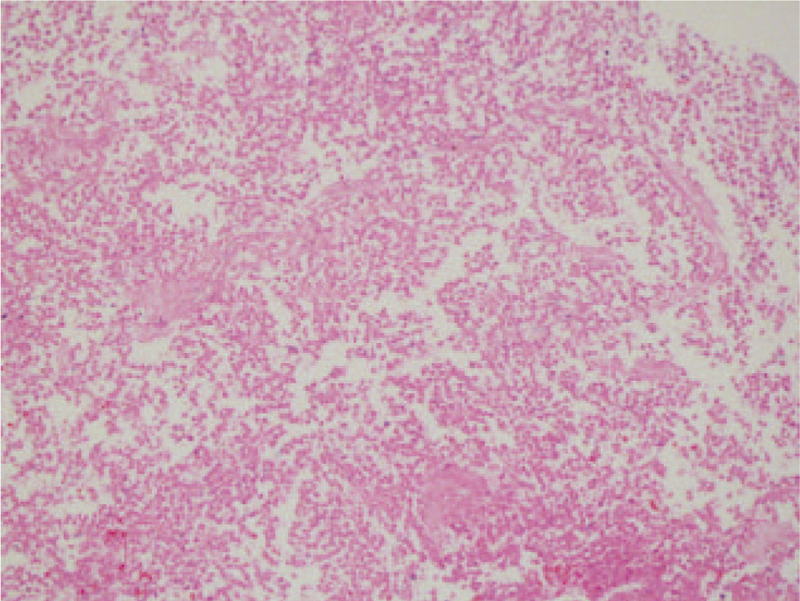
Pathologic image of biopsy from the vaginal mass (HE × 200). Small round-cells were uniform in size, with infiltration and extensive necrosis. HE = hematoxylin and eosin.

US images showed a hypoechoic or anechoic mass in the right lateral vaginal wall, measured 6.72 × 4.66 cm, with no obvious blood flow signal (Fig. 2A). Another well-defined heterogeneous hypoechoic mass with a little blood flow signal, measured 6.26 × 5.00 cm, was found in the medial aspect of posterior uterine wall (Fig. 2B). As to the vaginal mass, transvaginal ultrasound-guided puncture biopsy revealed that small round-cells were uniform in size, with infiltration and extensive necrosis (Figs. 2C and 3Figs. 2 and 3). CT scan and other related examination did not reveal any metastatic disease. Thus, the patient underwent hysterectomy, bilateral salpingo-oophorectomy, and tumors excision.

**Figure 4:**
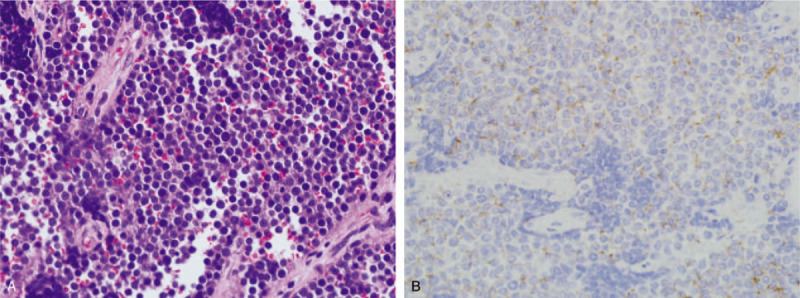
Histopathological and immunohistochemical (IHC) images of vaginal ES. (A) High power (400 × ) HE stain showed small cells with focal necrosis and mitotic activity. (B) Immunohistochemical findings presented positive expression of CD99 in the vaginal tumor cells (IHC × 400). ES = Ewing sarcoma, HE = hematoxylin and eosin.

**Figure 5:**
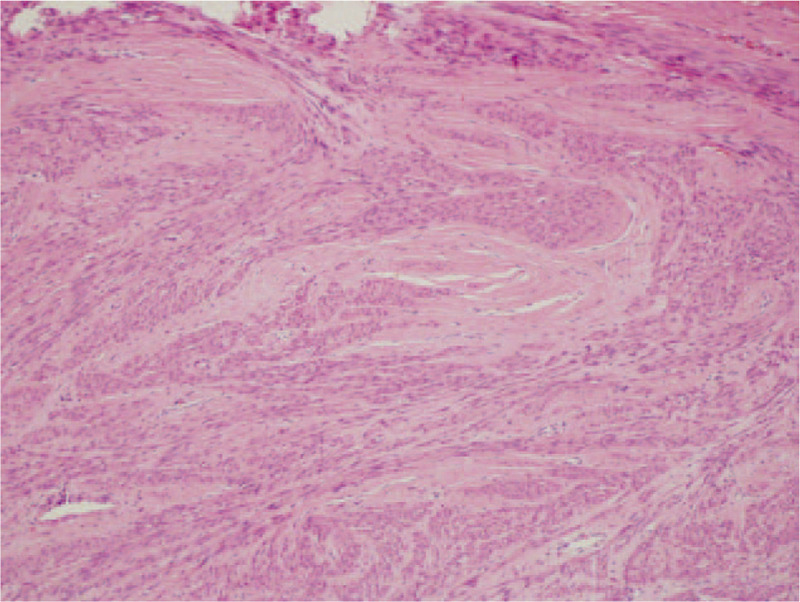
Postoperative pathologic image of the uterine fibroid (HE × 100). HE = hematoxylin and eosin.

Final surgical pathology of the 2 masses was confirmed as vaginal ES and uterine fibroid, respectively (Figs. 4A and 5Figs. 4 and 5). Moreover, the vaginal tumor involved her cervix and extending into the right parametrium but without detectable metastases, which should be definited as stage IIB according to the 8th edition of the AJCC criteria. Immunohistochemistry (IHC) showed diffuse membranous positivity for CD99 in vaginal lesion (Fig. 4B), strongly suggesting a diagnosis of Ewing's sarcoma. All resection margins were negative. Additionally, the patient was also managed with combination chemotherapy protocol (Lobaplatin 31.4 mg/m^2^, Etoposide 62.8 mg/m^2^). After 6 weeks of combination chemotherapy, there was a complete remission of the tumor by response evaluation criteria in solid tumors (RECIST) criteria. For >1-year follow-up, the ultrasound and other clinical examination demonstrated that the patient remained free of local recurrence and metastasis.

## 3 Discussion

Ewing sarcoma (ES) and primitive neuroectodermal tumor (PNET) are considered as single tumor category known as Ewing family of tumors (EFTs),^[2,8]^ which could be diagnosed in children and adults.^[1,2,4,9]^ Extra osseous ES mainly affects the paravertebral region, lower extremity, chest wall, retroperitoneum, pelvis, and hip,^[10]^ while the most common site of metastatic spread is lung.^[11]^ Pathologically, these tumors are often well demarcated with early involvement of adjacent soft tissues, in which necrosis and hemorrhage are common.^[4]^ As hormone-dependent tumor, uterine fibroids are benign tumors of smooth muscle cells separated by fibrous tissue.^[12]^ Up to 20% to 50% of uterine fibroids are symptomatic with symptoms, depending on the size, location, and degenerations.^[13]^ Different types of degeneration include hemorrhagic (red), cystic, calcified, myxomatous, lipomatous, and sarcomatous,^[12]^ and the degenerations are rarely pure occurring in fibroid.

The imaging findings of extraosseous ES is usually presented as a hypervascular soft tissue mass with areas of necrosis or hemorrhage. The tumors may also invade adjacent bones, but do not affect the bone marrow. Calcifications could appear in up to 25% of cases.^[14]^ There have been less case reports describing the imaging appearance of vaginal ES. On MRI examination, the tumors may be T2 isointense to hyperintense, T1 isointense to muscle, with heterogeneous contrast enhancement, while the tumor could demonstrate a heterogeneous ultrasonic appearance with few Doppler color flow.^[4]^ During ultrasound examination, fibroids usually appear as well-defined, solid, concentric, hypoechoic masses, with a variable amount of acoustic shadowing.^[7]^ MRI and computer tomography might be helpful in the diagnosis of uterine fibroid. A nondegenerated fibroid is usually as isointense to myometrium on T1 and hypointense on T2 sequences, and shows avid enhancement in MRI scans. Furthermore, different forms of degenerations may occur simultaneously in a given fibroid, thus the categorization on imaging may be based on the predominant imaging feature.^[12]^

Our case demonstrates a solid and cystic mass with obscure margin of the vaginal ES, while lesion's edge showed both restricted diffusion and marked enhancement in MRI images. Similar heterogeneous characteristics were seen on US, which manifested as a hypoechoic or anechoic mass, with no obvious blood flow signal inside. The MRI and US features could be interpreted by extensive area of necrosis or hemorrhage, which are the pathological features of vaginal ES. On MRI, the uterine fibroid of our case had smooth, well-defined margin, and heterogeneous enhancement, with a little contrast agent entering the mass, which may be owing to tumor degeneration.

Differential diagnosis for a patient with an aggressive pelvic (extraovarian) lesion should include rhabdomyosarcoma, extraskeletal ES, synovial sarcoma, malignant melanoma, and some less frequently carcinoma.^[15–17]^ On MRI, the proposed diagnostic imaging criteria for extraskeletal ES relied on the absence of adjacent bony invasion.^[14]^ Our case had no bone invasion, but the location of vaginal mass was considered to be indeterminate on MRI. In contrast, US could present the accurate location of the vaginal tumor. In comparison with MRI, US has several advantages, as it is widely available, well tolerated for patients, and it could be combined with a ultrasound-guided puncture biopsy,^[18]^ which is a safe, fast, and reliable method for pathological diagnosis.

Most uterine fibroids do not require treatment unless they are causing symptoms. After menopause, fibroids shrink, and it is unusual for them to cause problems. In those who have symptoms, uterine artery embolization and surgical options have similar outcomes with respect to satisfaction.^[19]^ A standard treatment guidelines available for vaginal ES is lacking due to rarity of its occurrence.^[2,20]^ Wide local excision followed by adjuvant chemotherapy and/or radiotherapy is recommended.^[1]^ The outcome for the tumor is primarily dependent on the presence of distant metastases. In the absence of detectable metastases, 70% of children and adolescents are cured with a combination of intensive, multiagent chemotherapy with surgery and/or radiotherapy.^[4,21]^ As to the present case, the patient was treated by hysterectomy, bilateral salpingo-oophorectomy and tumors excision, with the subsequent treatment of chemotherapy. The patient had remained free of local recurrence and metastasis for >1-year. Given the possibility of local recurrence, it is essential to have continuous close follow-up of the patient.

In conclusion, we report an extremely rare case of vaginal ES with uterine fibroid. Imaging is crucial for defining the extent of disease, evaluating response to treatment, and detecting recurrent/metastatic disease. Vaginal ES and uterine fibroid have certain imaging features in MRI and US, but not specific, while ultrasound-guided puncture biopsy plays a key role in confirming the diagnosis of vaginal ES.

## Acknowledgments

The protocol for the present retrospective study was approved by the Ethics Committee of Hubei Cancer Hospital, and informed consent was obtained from the patient.

## Author contributions

**Conceptualization:** Gaolong Weng, Fang Li.

**Investigation:** Shue Zeng, Hongbin Wang.

**Writing – original draft:** Maolin Xu, Fang Li.

**Writing – review & editing:** Yulin Liu, Shue Zeng.
